# Pandemic-Associated Shifts and Spatial Inequality in Public Attention to Pulmonary Function Testing in China: A 14-Year Baidu Index Study

**DOI:** 10.3390/healthcare14162640

**Published:** 2026-08-20

**Authors:** Cai Chen, Dedong Ma, Tao Jing

**Affiliations:** 1 State Key Laboratory of Environment Health (Incubation), Key Laboratory of Environment and Health, Ministry of Education, Key Laboratory of Environment and Health (Wuhan), Ministry of Environmental Protection, School of Public Health, Tongji Medical College, Huazhong University of Science and Technology, #13 Hangkong Road, Wuhan 430030, China; d202582315@hust.edu.cn; 2 Shandong Institute of Advanced Technology, Chinese Academy of Sciences, Jinan 250000, China; 3 Shandong Provincial Key Laboratory for Diagnosis and Treatment of Infectious Respiratory Diseases, Jinan 250000, China; 4 School of Medicine, Xizang University, No. 10 Zangda East Road, Lhasa 850000, China; 5 Xizang Autonomous Region Common Technology Innovation Center for Medical Equipment on the Plateau, Lhasa 850000, China

**Keywords:** pulmonary function test, Baidu Index, infodemiology, public attention, COVID-19, health information-seeking behavior, spatial inequality

## Abstract

**Background/Objectives:** Pulmonary function tests (PFTs), especially spirometry, are important for chronic respiratory disease diagnosis and management, but Baidu-based online search interest in PFT-related information remains insufficiently characterized. This study examined long-term Baidu search activity for a selected PFT-related keyword in China from 2011 to 2024, with attention to pandemic-period changes, provincial variation, spatial inequality, and associated socioeconomic factors. **Methods:** We analyzed Baidu Index data from 1 January 2011 to 31 December 2024. The selected PFT-related keyword, “pulmonary function instrument,” was compared with “blood pressure monitor” and “blood glucose meter.” Daily Baidu Index values were aggregated into monthly and annual series. Temporal trends, relative search-intensity ratios, interrupted time-series analysis, inequality indices, fixed-effects regression, subgroup heterogeneity, and convergence dynamics were assessed. **Results:** PFT-related Baidu search activity remained lower than that for blood pressure and glucose monitoring, with relative search-intensity ratios below 0.35. Descriptively, PFT-related searches increased during 2020–2022; however, the re-specified seasonality-adjusted ITSA using unsmoothed monthly observations did not provide strong statistical evidence for an immediate level change at pandemic onset or a sustained slope change. Provincial differences persisted, with higher values generally observed in several eastern coastal provinces. Inequality indices were lower on average during 2020–2022 but fluctuated over time, and beta-convergence analyses provided limited statistical evidence of systematic catch-up among initially low-index provinces. Urbanization, per capita GDP, and population aging were positively associated with PFT-related Baidu Index values, while subgroup findings were interpreted as exploratory ecological associations. **Conclusions:** PFT-related Baidu search activity in China remained lower than search activity for blood pressure and glucose monitoring and showed persistent provincial variation. These findings suggest lower visibility of PFT-related information within the Baidu-based online environment.

## 1. Introduction

Chronic respiratory diseases, particularly chronic obstructive pulmonary disease (COPD) and asthma, represent a major and growing global public health burden. COPD is characterized by progressive airflow limitation, recurrent exacerbations, and high mortality, and is now recognized as one of the leading causes of death worldwide. Recent epidemiological evidence estimates that approximately 391.9 million individuals aged 30–79 years are affected globally, with a disproportionate burden in low- and middle-income regions [1]. In the United States, COPD prevalence has remained substantial over the past decade, with persistent disparities across age, smoking status, and residential setting [2]. In China, the disease burden is even more pronounced, with an estimated prevalence of 8.6% among adults aged ≥20 years, rising to more than 27% in individuals over 60 years, corresponding to nearly 100 million patients nationwide [3]. Regional disparities are also evident, with particularly high prevalence reported in rural western China [4].

Pulmonary function tests (PFTs), especially spirometry, are widely recognized as the gold standard for the diagnosis and functional assessment of chronic respiratory diseases. Key parameters such as forced expiratory volume in one second (FEV_1_) and forced vital capacity (FVC) provide objective measures for diagnosing airflow limitation, assessing disease severity, and evaluating therapeutic response [5,6]. Despite their clinical value, PFTs remain substantially underutilized in both developed and developing healthcare systems. Evidence from large cohort studies indicates that fewer than half of patients with asthma or COPD undergo spirometric testing around the time of diagnosis [7,8,9]. Even in high-income settings, testing rates remain suboptimal, with significant disparities across age groups, socioeconomic status, and healthcare access.

This underutilization has critical clinical consequences. The absence of objective lung function assessment contributes to both underdiagnosis and misclassification of respiratory diseases. For example, only 23.4% of patients with asthma in the China Pulmonary Health study had undergone prior lung function testing [10], and similar gaps have been reported in pediatric populations in Europe and North America [11]. These findings collectively highlight a persistent disconnect between clinical guidelines and real-world implementation.

Beyond clinical practice, public awareness and health-seeking behavior play a fundamental role in shaping early diagnosis and preventive care. Although national health policies in China have explicitly emphasized increasing awareness of COPD and promoting annual lung function testing among high-risk populations since 2019, actual uptake remains limited, with many patients only receiving testing at advanced disease stages. In this context, the rapid expansion of internet-based health information-seeking behavior offers a novel opportunity to assess population-level awareness.

Recent studies have demonstrated the feasibility of using search engine data as a proxy for public health interest and disease awareness. For instance, Baidu Index data have been successfully used to characterize asthma trends [12], seasonal respiratory symptom patterns [13], and other population health behaviors [14]. However, existing PFT-related research has mainly focused on clinical indications, diagnostic accuracy, disease management, and underutilization in healthcare settings, while population-level online attention to PFTs remains insufficiently studied. In particular, little is known about how public search interest in PFTs has changed over a long-term period spanning the pre-pandemic, pandemic, and post-pandemic policy-transition stages, or how such attention varies across regions in China. To address this gap, this study conducts a 14-year longitudinal analysis of Baidu Index data in China from 1 January 2011 to 31 December 2024. By integrating multi-phase interrupted time series analysis and fixed-effects panel regression, we aim to (1) quantify temporal and spatial trends in public attention to PFTs, (2) evaluate the impact of the COVID-19 pandemic as a population-level shock, and (3) identify socioeconomic determinants and regional heterogeneity in health information-seeking behavior. Furthermore, by benchmarking PFT-related Baidu search activity against searches for blood pressure and glucose monitoring, we provide a comparative framework for describing relative online attention to different health-monitoring topics. Because Baidu Index reflects online search behavior rather than actual clinical utilization or healthcare need, these comparisons were interpreted as Baidu-based information-seeking patterns rather than direct measures of PFT uptake or unmet respiratory healthcare demand.

## 2. Methods

### 2.1. Data Source and Study Design

This study adopted a longitudinal observational framework to explore nationwide public attention toward pulmonary function testing across China from 1 January 2011 to 31 December 2024. We retrieved aggregated internet search data from the Baidu Index, which quantifies population-level search intensity of given keywords among Chinese netizens. The Baidu Index is an online search analytics platform based on aggregated Baidu search behavior. The Baidu Search Index uses user-specified keywords as the statistical unit and reports platform-generated index values calculated from weighted search frequency in Baidu web search, rather than absolute query counts. Baidu Index distinguishes PC and mobile search sources and also provides an overall search index. Because the exact weighting and scaling algorithm is proprietary and not publicly disclosed, Baidu Index values should be interpreted as platform-specific indicators of relative online search intensity. They do not directly measure clinical service utilization, disease incidence, healthcare demand, public awareness, respiratory health need, or individual-level behavior. This metric has been widely used in infodemiology research as a proxy for online health information-seeking behavior, but not as a direct measure of healthcare utilization or clinical demand.

Three Chinese Baidu Index keyword entries were used in the primary analysis: “肺功能仪” (translated as “pulmonary function instrument”), “血压计” (“blood pressure monitor”), and “血糖仪” (“blood glucose meter”). These terms were entered as Baidu Index keyword entries rather than manually combined keyword groups or broad Baidu topic categories. No custom aggregation of multiple respiratory keywords was used in the primary analysis. The selected PFT-related keyword, “肺功能仪,” is device-oriented and may be searched by multiple user groups, including lay users interested in lung function assessment, healthcare professionals, institutions, purchasers, and medical-device suppliers. Therefore, this keyword was interpreted as an imperfect but stable operational proxy for Baidu-based online search interest in PFT-related equipment and lung function assessment information, rather than as a direct measure of general public awareness of spirometry, PFT appointment demand, clinical utilization, or actual testing uptake. The comparator terms “血压计” and “血糖仪” are widely recognized in China as household self-monitoring devices for blood pressure and glucose monitoring. Accordingly, the comparison between PFT-related searches and these two comparator terms was used to describe relative Baidu-based search intensity across health-monitoring topics, not to imply that the three keywords represent equivalent testing modalities or the same type of healthcare behavior. Given the lack of continuous long-term search records for direct or related respiratory-function keywords, including lung function, pulmonary function, pulmonary function test, pulmonary function examination, pulmonary function measurement, spirometer, and vital capacity meter in the Baidu Index database, we adopted “pulmonary function instrument” as a stable alternative indicator to ensure data integrity throughout the study period. We acknowledge that searches for “pulmonary function instrument” may reflect multiple forms of attention, including product information seeking, purchasing interest, institutional procurement, or commercially oriented searches, rather than clinical service demand alone. Therefore, this keyword was interpreted as a proxy for online attention to PFT-related equipment and lung function assessment, not as a direct measure of PFT appointment demand, clinical utilization, or actual testing uptake. We also acknowledge that this keyword differs in modality from home blood pressure and glucose monitoring devices. Pulmonary function testing, especially spirometry, is usually performed in clinical or professionally supervised settings, whereas blood pressure and glucose monitoring can be self-administered at home. Therefore, the selected PFT-related keyword was used to capture relative online attention to respiratory function testing rather than to represent an equivalent home-monitoring behavior.

For each keyword, we retrieved daily Baidu Search Index values from 1 January 2011 to 31 December 2024 at the national and provincial levels, covering all 31 provincial-level administrative regions in mainland China. The primary analyses used the overall search-source category, corresponding to combined PC and mobile search activity. We did not apply additional min–max normalization or z-score normalization to the Baidu Index values before the primary temporal, spatial inequality, or convergence analyses. The term “index value” refers to the platform-generated Baidu Index value. Monthly and annual values were calculated by aggregating daily overall Baidu Index values using arithmetic means. Therefore, the analyses compare Baidu-normalized, platform-specific search intensity rather than absolute query counts, population-adjusted search rates, or clinical demand. Monthly and annual Baidu Index values were calculated as follows:

(1) $$BI_{k,m}=\frac{1}{n_{m}}\sum_{d\in m}BI_{k,d}^{all},$$

(2) $$BI_{k,y}=\frac{1}{n_{y}}\sum_{d\in y}BI_{k,d}^{all},$$

where $BI_{k,d}^{all}$ denotes the daily overall Baidu Index value for keyword *k* on day *d*, and $n_{m}$ and $n_{y}$ denote the number of observed days in month *m* and year *y*, respectively.

Data quality checks were performed before aggregation. Daily records were checked for duplicate dates, missing dates, missing index values, and zero values. Zero values were retained as valid Baidu Index values because they may reflect very low platform-recorded search intensity. For the relative search-intensity ratios, denominator values were checked to avoid undefined ratios. Extreme daily peaks were not removed or winsorized, because they may represent genuine short-term bursts of online search interest. Monthly aggregation and the use of Newey–West robust standard errors in the ITSA were used to reduce the influence of short-term volatility.

### 2.2. Spatiotemporal Trend and Relative Attention Analysis

We first constructed continuous time-series curves of keyword search intensity spanning 2011 to 2024 to depict long-term temporal trends. Moving averages were used only for descriptive visualization of long-term trends. All inferential interrupted time-series analyses were conducted using unsmoothed monthly Baidu Index observations. To compare relative Baidu-based search intensity across different health-monitoring topics, we built two relative search ratios:

(3) $$R_{\mathrm{PFT}/\mathrm{BP},t}=\frac{BI_{\mathrm{PFT},t}}{BI_{\mathrm{BP},t}},\;R_{\mathrm{PFT}/\mathrm{BG},t}=\frac{BI_{\mathrm{PFT},t}}{BI_{\mathrm{BG},t}},$$

where $BI_{\mathrm{PFT},t}$ denotes the search intensity of pulmonary function testing equipment at time *t*, $BI_{\mathrm{BP},t}$ denotes the search intensity of blood pressure monitor, and $BI_{\mathrm{BG},t}$ denotes the search intensity of blood glucose meter. These two ratios were used as benchmarking indicators to compare relative online attention to respiratory function testing with attention to more familiar chronic disease monitoring behaviors. Because blood pressure and glucose monitoring are commonly self-administered at home, whereas pulmonary function testing is mainly a clinical procedure, these ratios were not interpreted as direct comparisons between equivalent testing modalities.

For spatial pattern analysis, we used normalized provincial Baidu Index values to generate choropleth maps. The visualization described the evolution of interprovincial variation in PFT-related Baidu search intensity over time.

### 2.3. Multi-Phase Interrupted Time Series Analysis

We implemented a multi-phase interrupted time-series analysis (ITSA) to evaluate changes in PFT-related search activity before, during, and after the COVID-19 pandemic. In response to concerns that pre-model smoothing may alter level and slope estimates, the primary ITSA was re-specified using unsmoothed monthly Baidu Index observations. The analysis included 168 monthly observations from January 2011 to December 2024.

The whole observation window was divided into three consecutive stages: (1) the pre-pandemic period, January 2011–December 2019; (2) the pandemic period, January 2020–December 2022; and (3) the post-pandemic recovery period, January 2023–December 2024. January 2023 was selected as the starting point of the post-pandemic recovery period because it immediately followed the major adjustment of China’s COVID-19 prevention and control policy in December 2022, when large-scale zero-COVID control measures were relaxed. Therefore, this cut-off was defined according to a major public health policy transition rather than chronological convenience alone.

The segmented regression model [15,16] was specified as follows:

(4) $$Y_{t}=\beta_{0}+\beta_{1}T_{t}+\beta_{2}D_{1t}+\beta_{3}P_{1t}+\beta_{4}D_{2t}+\beta_{5}P_{2t}+\sum_{m=2}^{12}\gamma_{m}M_{mt}+\varepsilon_{t},$$

where $Y_{t}$ denotes the unsmoothed monthly Baidu Index value at month *t*; $T_{t}$ is a continuous time counter starting from January 2011; $D_{1t}$ is coded as 1 during January 2020–December 2022 and 0 otherwise; $P_{1t}$ is coded as 1–36 during January 2020–December 2022 and 0 otherwise; $D_{2t}$ is coded as 1 from January 2023 onward and 0 otherwise; $P_{2t}$ is coded as 1–24 from January 2023 onward and 0 otherwise; $M_{mt}$ represents calendar-month fixed effects, with January as the reference month; and $\varepsilon_{t}$ is the random error term. The coefficients were estimated using ordinary least squares. Calendar-month fixed effects were included to explicitly adjust for seasonality. Newey–West robust standard errors with a lag length of 12 were used to account for autocorrelation and heteroskedasticity in monthly data, because a 12-month lag captures potential annual seasonal dependence. We reported all coefficients, standard errors, 95% confidence intervals, and *p*-values. Model diagnostics included the Durbin–Watson statistic, Ljung–Box test, Breusch–Pagan test, Jarque–Bera test, adjusted $R^{2}$, and variance inflation factor. Sensitivity analyses were further conducted using alternative Newey–West lag lengths and alternative interruption dates.

### 2.4. Fixed-Effects Panel Regression Model

We established a provincial annual panel dataset to examine socioeconomic factors associated with PFT-related Baidu Index values. The dependent variable was the annual mean provincial Baidu Index value for the selected PFT-related keyword, calculated from daily overall Baidu Index values. The explanatory variables included per capita gross domestic product (GDP), urbanization rate, educational attainment, population aging ratio, and health workforce density. Per capita GDP was used to describe regional economic development; urbanization rate was defined as the proportion of the urban resident population; educational attainment was defined as the share of the population with college education or above; population aging ratio was defined as the percentage of residents aged 65 years or older; and health workforce density was measured as the number of health technicians per 1000 residents. Socioeconomic variables were obtained from official provincial statistical sources. Per capita GDP, urbanization rate, educational attainment, and population aging ratio were obtained from the China Statistical Yearbook and corresponding provincial statistical yearbooks. Health workforce density was obtained from the China Health Statistics Yearbook and provincial health statistical yearbooks. All variables were matched by province and year to the annual provincial Baidu Index dataset. The two-way fixed-effects model was specified as follows:

(5) $$Y_{it}=\alpha +\beta X_{it}+\mu_{i}+\lambda_{t}+\varepsilon_{it},$$

where $Y_{it}$ denotes the annual PFT-related Baidu Index value for province *i* in year *t*; $X_{it}$ is a vector of socioeconomic covariates; $\mu_{i}$ represents province fixed effects; $\lambda_{t}$ represents year fixed effects; and $\varepsilon_{it}$ is the idiosyncratic error term.

The full provincial annual panel included 31 provinces over 14 years, corresponding to 434 province-year observations. Because health workforce density had missing or non-numeric values in some province-year records, the primary fixed-effects regression was estimated using complete-case analysis, resulting in 424 observations. Missing values were not imputed. The primary model used annual provincial variables in their original units. To facilitate comparison across variables measured on different scales, standardized coefficients were also estimated by applying z-score standardization to both the dependent variable and each independent variable. Therefore, both unstandardized and standardized coefficients were reported. The primary regression reported heteroskedasticity-consistent robust standard errors. As a sensitivity analysis, the model was also examined using province-clustered standard errors to account for potential within-province correlation over time. Pairwise correlation coefficients and variance inflation factors were calculated to assess correlation and multicollinearity among socioeconomic variables. These diagnostic results are reported in the Supplementary Materials. To evaluate whether socioeconomic characteristics showed delayed associations with PFT-related Baidu Index values, we further conducted sensitivity analyses using one-year lagged socioeconomic variables. These lagged models were treated as sensitivity analyses rather than primary causal models.

The two-way fixed-effects specification adjusts for time-invariant provincial characteristics and common nationwide year-specific shocks. However, it cannot eliminate bias from unmeasured time-varying confounders, such as regional internet penetration, Baidu platform use, media exposure, respiratory disease burden, healthcare policy changes, or changes in clinical service availability. Therefore, the fixed-effects estimates were interpreted as ecological associations rather than causal effects.

### 2.5. Heterogeneity Analysis

We conducted stratified subgroup analyses to examine whether the association between provincial characteristics and PFT-related Baidu Index values differed across regional development contexts. Provinces were grouped according to three prespecified stratification criteria: (1) geographic division, including Eastern, Central, and Western China; (2) economic development level, with high-income and low-income provinces defined according to the median of study-period mean per capita GDP; and (3) urbanization level, with high-urbanization and low-urbanization provinces defined according to the median of study-period mean urbanization rate. Within each subgroup, we re-estimated the two-way fixed-effects regression model using the same dependent variable and covariates as in the main model. The subgroup analysis focused on the association between population aging ratio and PFT-related Baidu Index values because population aging was considered clinically relevant to respiratory health information seeking. For each subgroup, standardized coefficients, robust standard errors, 95% confidence intervals, *p*-values, numbers of observations, and adjusted $R^{2}$ values were reported.

Median-based dichotomization was used only for exploratory stratified description because it provides a transparent and interpretable way to compare provinces with relatively higher versus lower economic development or urbanization levels. We acknowledge that dichotomizing continuous variables may result in information loss. Therefore, subgroup findings were interpreted cautiously and were not treated as definitive evidence of effect modification. Because subgroup analyses involved multiple exploratory comparisons, nominal *p*-values were interpreted cautiously. No formal multiple-testing correction was applied; therefore, subgroup results should be considered hypothesis-generating. Where between-group differences were examined, they were interpreted as exploratory evidence of heterogeneity rather than causal interaction effects.

### 2.6. Spatial Convergence Analysis

We further examined whether provincial differences in PFT-related Baidu Index values showed evidence of spatial convergence over time. Two complementary approaches were used: σ-convergence and β-convergence. σ-convergence was defined as a decline over time in the cross-provincial dispersion of PFT-related Baidu Index values. It was measured using the standard deviation of log-transformed provincial Baidu Index values:

(6) $$\sigma_{t}=SD_{i}[\log(BI_{it}+1)],$$

where $BI_{it}$ denotes the PFT-related Baidu Index value for province *i* in year *t*. The transformation log(BI+1) was used to reduce skewness and accommodate zero values. β-convergence was examined by regressing annualized log growth in PFT-related Baidu Index values from 2011 to 2024 on the initial log-transformed Baidu Index value in 2011:

(7) $$g_{i}=\alpha +\beta \log\left(BI_{i,2011}+1\right)+\varepsilon_{i},$$

where $g_{i}$ denotes the annualized log growth rate:

(8) $$g_{i}=\frac{\log\left(BI_{i,2024}+1\right)-\log\left(BI_{i,2011}+1\right)}{13}.$$

A negative β coefficient indicates that provinces with lower initial Baidu Index values tended to have higher subsequent growth, which is consistent with convergence. However, a negative coefficient was interpreted as evidence of convergence only when statistically supported. Statistically nonsignificant negative coefficients were interpreted as limited or insufficient statistical evidence of convergence. The β-convergence regressions were estimated using ordinary least squares. No population weighting was applied because Baidu Index values are platform-generated index values rather than raw search counts or per-capita rates. Standard errors, 95% confidence intervals, *p*-values, and $R^{2}$ values were reported. The full-sample convergence model included all 31 provinces. Region-specific analyses were conducted separately for Eastern, Central, and Western China. Because these regional models involved relatively small numbers of provinces, the region-specific convergence results were interpreted cautiously and treated as exploratory.

### 2.7. Statistical Software

All statistical analyses were completed using R version 4.3.2 and Python version 3.11.7. Provincial spatial distribution maps were produced using Python version 3.11.7 with GeoPandas version 0.14.4, Shapely version 2.0.4, and Matplotlib version 3.8.4. A two-sided statistical significance threshold of p<0.05 was used for all hypothesis tests.

## 3. Results

### 3.1. Spatiotemporal Evolution Under Pandemic Shock

**Figure 1:**
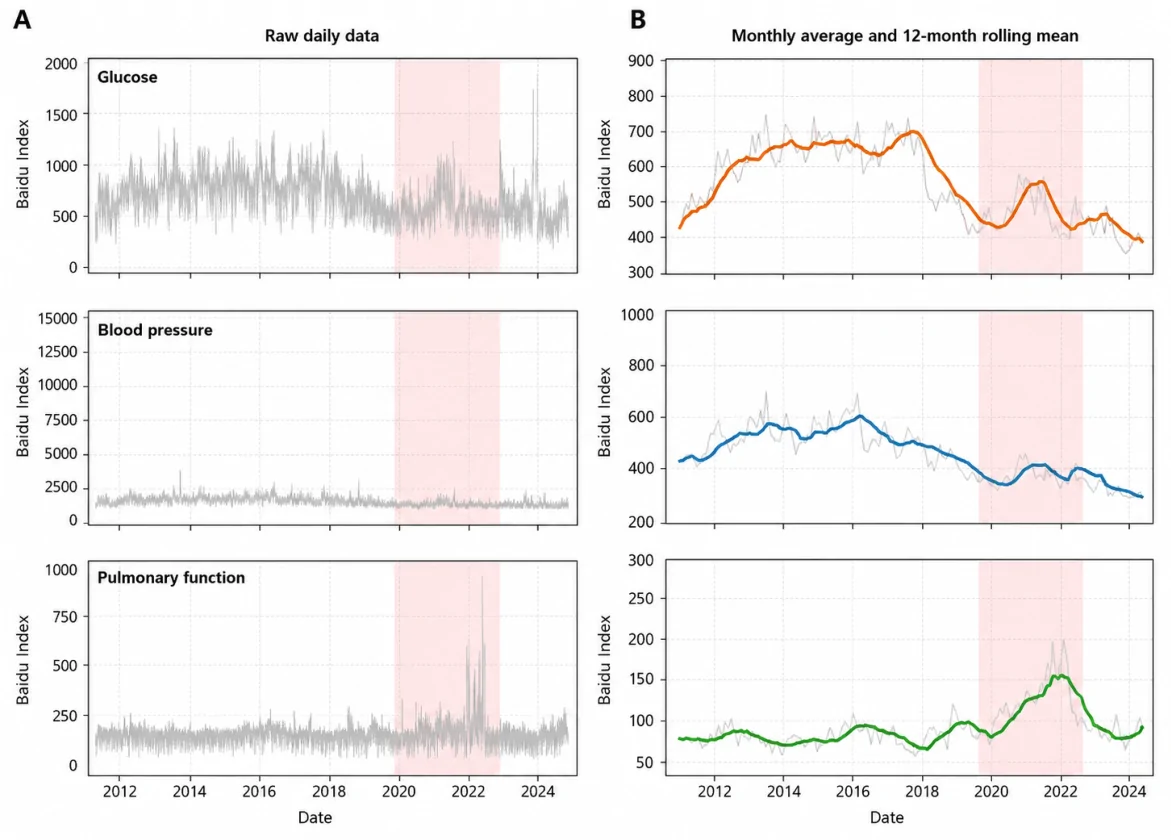
National search trends of three health-related search terms in China, 2011–2024. (**A**) Raw daily overall Baidu Index values for “blood glucose meter,” “blood pressure monitor,” and “pulmonary function instrument” from 2011 to 2024. (**B**) Monthly averages and 12-month rolling means calculated from the raw daily overall Baidu Index values. Grey lines indicate monthly average values, and colored lines indicate smoothed 12-month rolling means. The shaded area denotes the COVID-19 pandemic period from 2020 to 2022.

Figure 1 shows the national temporal trends of the three keyword series from 2011 to 2024. PFT-related Baidu search activity remained consistently lower than search activity for blood pressure and glucose monitoring across the entire observation period. However, the three keywords showed different temporal trajectories. Blood pressure monitor- and glucose meter-related searches reached earlier peaks and declined in later years, whereas PFT-related search activity remained at a lower baseline and showed a descriptive increase during 2020–2022.

**Figure 2:**
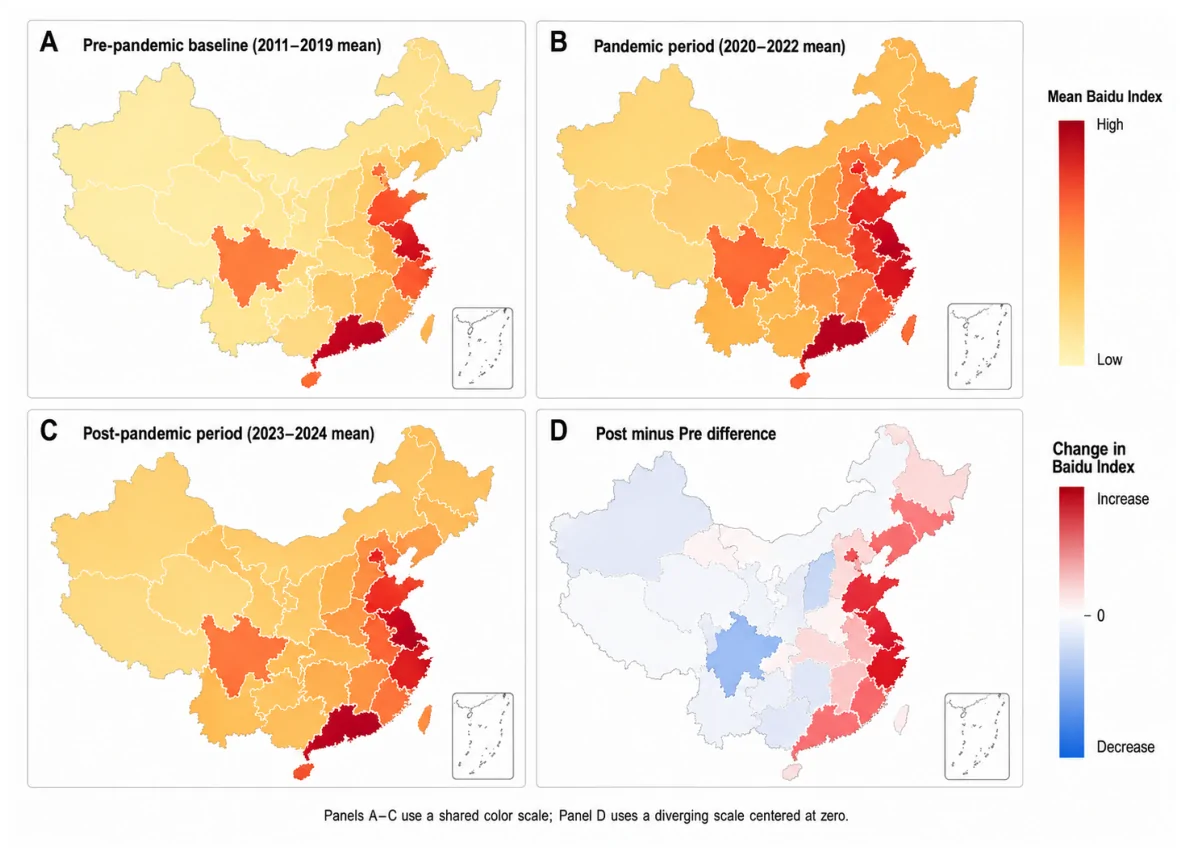
Spatiotemporal decomposition of pulmonary function test-related search activity across China. (**A**) Mean provincial Baidu Index during the pre-pandemic baseline period, 2011–2019. (**B**) Mean provincial Baidu Index during the pandemic period, 2020–2022. (**C**) Mean provincial Baidu Index during the post-pandemic period, 2023–2024. (**D**) Difference in provincial Baidu Index between the post-pandemic and pre-pandemic periods. Panels (**A**–**C**) use a shared sequential color scale, with darker colors indicating higher search activity. Panel (**D**) uses a diverging color scale centered at zero, where red indicates an increase and blue indicates a decrease in search activity. The small black-and-white inset in the lower-right corner of each panel is a standard South China Sea islands inset included for cartographic completeness and was not used in the statistical analysis.

Spatially, Figure 2 shows a persistent east–west gradient in PFT-related Baidu search intensity. Eastern coastal provinces, including Guangdong, Jiangsu, and Zhejiang, generally exhibited higher search intensity, whereas many central and western provinces showed relatively lower values. The difference map in Figure 2D indicates heterogeneous provincial changes between the post-transition and pre-pandemic periods. Increases were more evident in several eastern and coastal provinces, while some central and western provinces showed limited increases or decreases. Therefore, the spatial pattern should be interpreted as heterogeneous provincial variation in Baidu-based search interest rather than as a uniform nationwide increase or a western-region-dominant increase.

**Figure 3:**
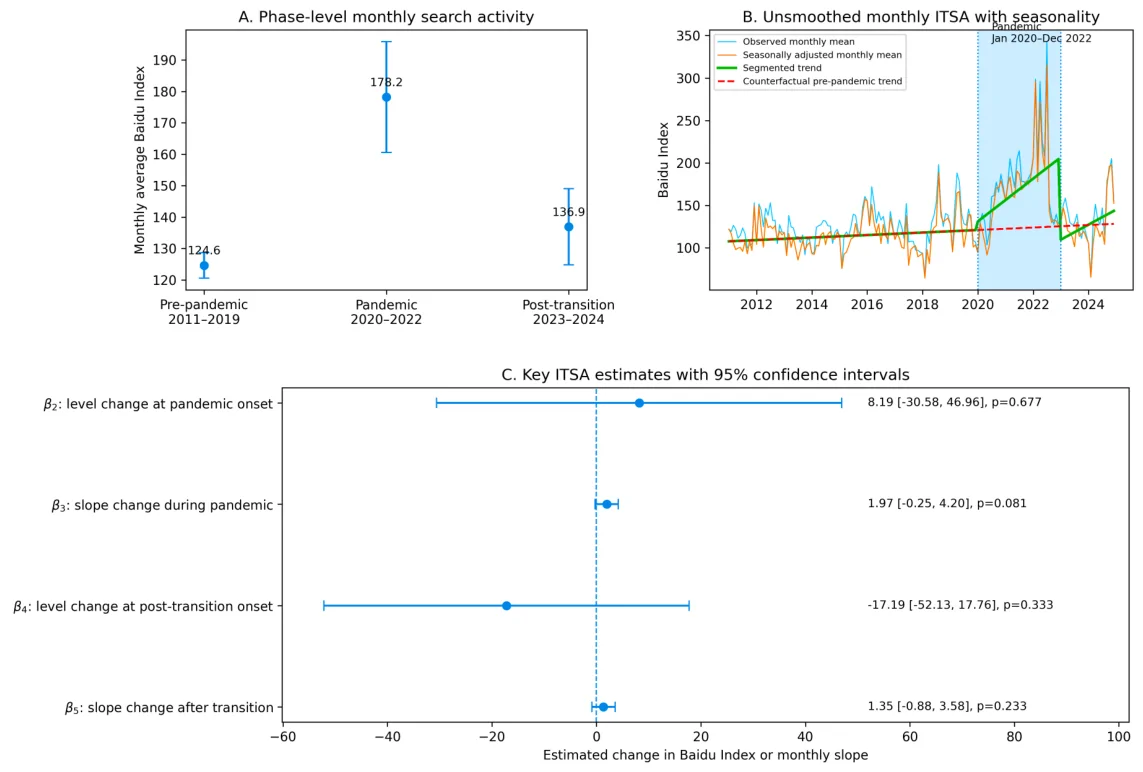
Re-specified interrupted time-series analysis of pulmonary function test-related search activity. (**A**) Phase-level monthly search activity across the pre-pandemic, pandemic, and post-transition periods. Points indicate phase means, and error bars indicate 95% confidence intervals. (**B**) Interrupted time-series model using unsmoothed monthly Baidu Index observations, with calendar-month fixed effects for seasonality and Newey–West robust standard errors. The shaded area denotes the COVID-19 pandemic period from January 2020 to December 2022, and the vertical reference lines indicate January 2020 and January 2023. (**C**) Estimated level and slope changes from the re-specified ITSA model. Points represent estimates, and horizontal lines indicate 95% confidence intervals. $\beta_{2}$ represents the immediate level change at pandemic onset, $\beta_{3}$ represents the slope change during the pandemic period, $\beta_{4}$ represents the immediate level change at post-transition onset, and $\beta_{5}$ represents the slope change after the transition.

The re-specified ITSA results are visualized in Figure 3 and reported numerically in Table 1. The re-specified ITSA model was based on 168 unsmoothed monthly observations from January 2011 to December 2024. After adjustment for calendar-month fixed effects and Newey–West robust standard errors with a lag length of 12, the baseline pre-pandemic slope was not statistically significant ($\beta_{1}=0.12$ per month, 95% CI: −0.08 to 0.33, p=0.237). The immediate level change at the onset of the pandemic was positive but not statistically significant ($\beta_{2}=8.19$, 95% CI: −30.58 to 46.96, p=0.677). The pandemic-period slope change was also positive but did not reach conventional statistical significance ($\beta_{3}=1.97$ per month, 95% CI: −0.25 to 4.20, p=0.081). After the transition to the post-pandemic period in January 2023, the immediate level change was negative but not statistically significant ($\beta_{4}=-17.19$, 95% CI: −52.13 to 17.76, p=0.333), and the post-transition slope change was also not statistically significant ($\beta_{5}=1.35$ per month, 95% CI: −0.88 to 3.58, p=0.233).

The estimated phase-specific slopes were 0.12 per month during the pre-pandemic period (95% CI: −0.08 to 0.33, p=0.237), 2.10 per month during the pandemic period (95% CI: −0.11 to 4.30, p=0.062), and 1.48 per month during the post-transition period (95% CI: −0.74 to 3.69, p=0.191). These results indicate that PFT-related search activity showed a descriptive elevation and a positive trend during the pandemic period, but the primary seasonality-adjusted ITSA did not provide strong statistical evidence for an immediate level change or a sustained slope change.

### 3.2. Relative Search Intensity and Spatial Variation in Baidu-Based Attention

**Figure 4:**
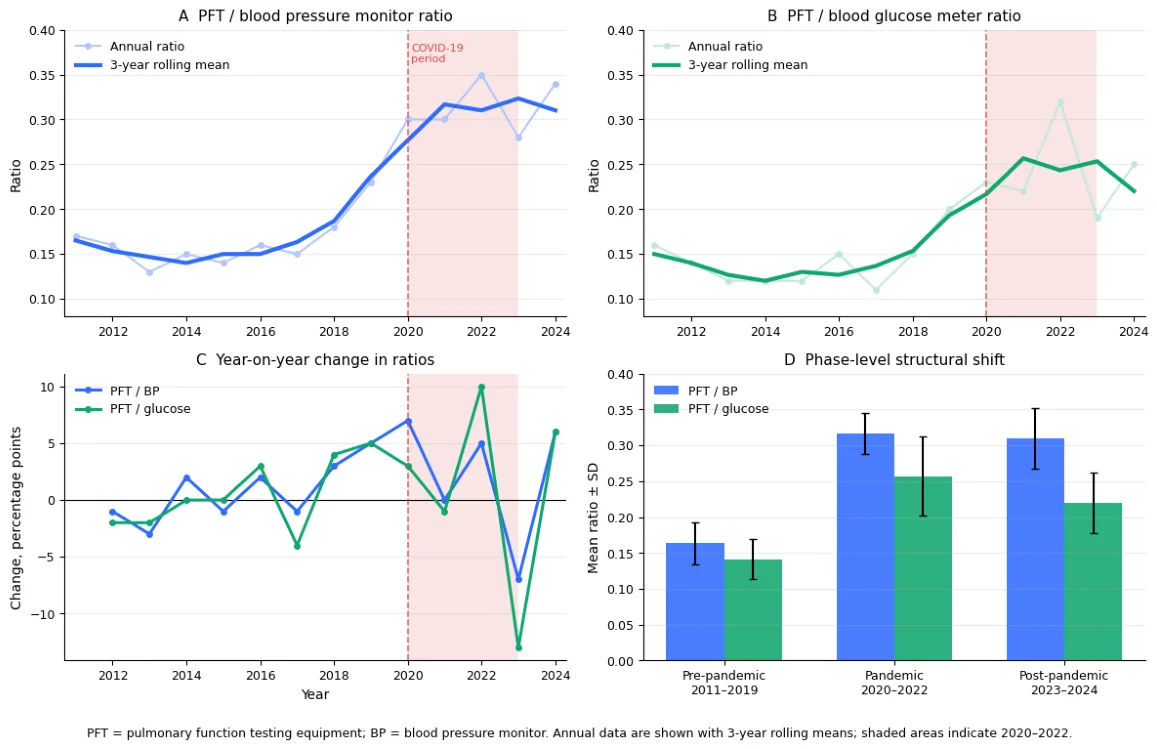
Relative search activity of pulmonary function testing compared with routine health monitoring, 2011–2024. (**A**) Annual ratio of pulmonary function testing-related searches to blood pressure monitor-related searches, with the 3-year rolling mean. (**B**) Annual ratio of pulmonary function testing-related searches to blood glucose meter-related searches, with the 3-year rolling mean. (**C**) Year-on-year changes in the two relative attention ratios. (**D**) Phase-level mean ratios across the pre-pandemic, pandemic, and post-pandemic periods, with error bars indicating standard deviations. Shaded areas indicate the COVID-19 pandemic period from 2020 to 2022. PFT denotes pulmonary function testing; BP denotes blood pressure monitor.

A persistent difference in relative Baidu search intensity was observed between PFT-related terms and the two comparator terms. As shown in Figure 4, the ratio of PFT-related searches to blood pressure monitor- and glucose meter-related searches remained below 0.35 throughout 2011–2024. These ratios should be interpreted as descriptive benchmarking indicators of relative search intensity, rather than as direct comparisons of equivalent household monitoring behaviors. Blood pressure monitors and glucose meters are widely recognized household self-monitoring devices, whereas conventional spirometry and pulmonary function testing are usually performed in healthcare or professionally supervised settings. Therefore, the lower PFT/BP and PFT/glucose ratios may reflect differences in device ownership, testing setting, cost, accessibility, and clinical indications, rather than deficient public recognition of respiratory health alone. These findings indicate that PFT-related online attention remained lower than attention to more familiar monitoring tools, although part of this difference may reflect lower home-use accessibility and stronger dependence on clinical service pathways rather than public awareness alone.

**Figure 5:**
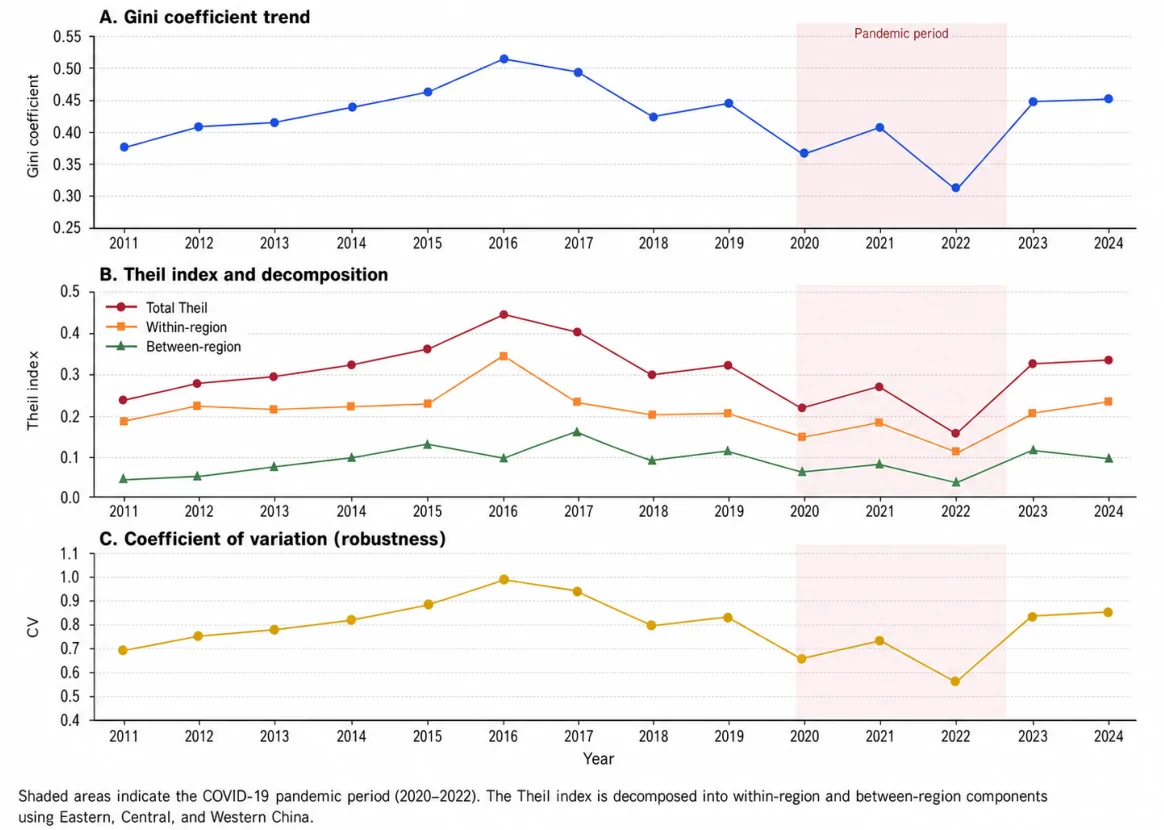
Temporal evolution of spatial inequality in public attention to pulmonary function testing in China. (**A**) Annual trend in the Gini coefficient of provincial pulmonary function testing-related search activity. (**B**) Theil index and its decomposition into within-region and between-region components based on Eastern, Central, and Western China. (**C**) Coefficient of variation as a robustness measure of spatial dispersion. Shaded areas indicate the COVID-19 pandemic period from 2020 to 2022.

Spatial inequality was further evaluated using the Gini coefficient, Theil index, and coefficient of variation. As shown in Figure 5 and Panel A of Table 2, the period-averaged Gini coefficient was 0.443 ± 0.043 during the pre-pandemic period, 0.365 ± 0.047 during the pandemic period, and 0.450 ± 0.003 during the post-pandemic period. The corresponding Theil index values were 0.331 ± 0.065, 0.222 ± 0.055, and 0.333 ± 0.006, respectively. The coefficient of variation showed a similar period-averaged pattern, with values of 0.832 ± 0.094, 0.656 ± 0.085, and 0.846 ± 0.009 across the three periods. These results indicate that average spatial inequality indices were lower during 2020–2022 than during the pre-pandemic and post-pandemic periods, although Figure 5 also shows short-term within-period fluctuations.

Overall, these results indicate two descriptive patterns in Baidu-based search activity: lower relative search activity for PFT-related terms compared with blood pressure and glucose monitoring terms, and persistent provincial variation in PFT-related Baidu Index values over time. These patterns should not be interpreted as direct evidence of clinical utilization, unmet healthcare need, or actual respiratory disease burden.

### 3.3. Socioeconomic Associations, Subgroup Heterogeneity, and Convergence Dynamics

**Figure 6:**
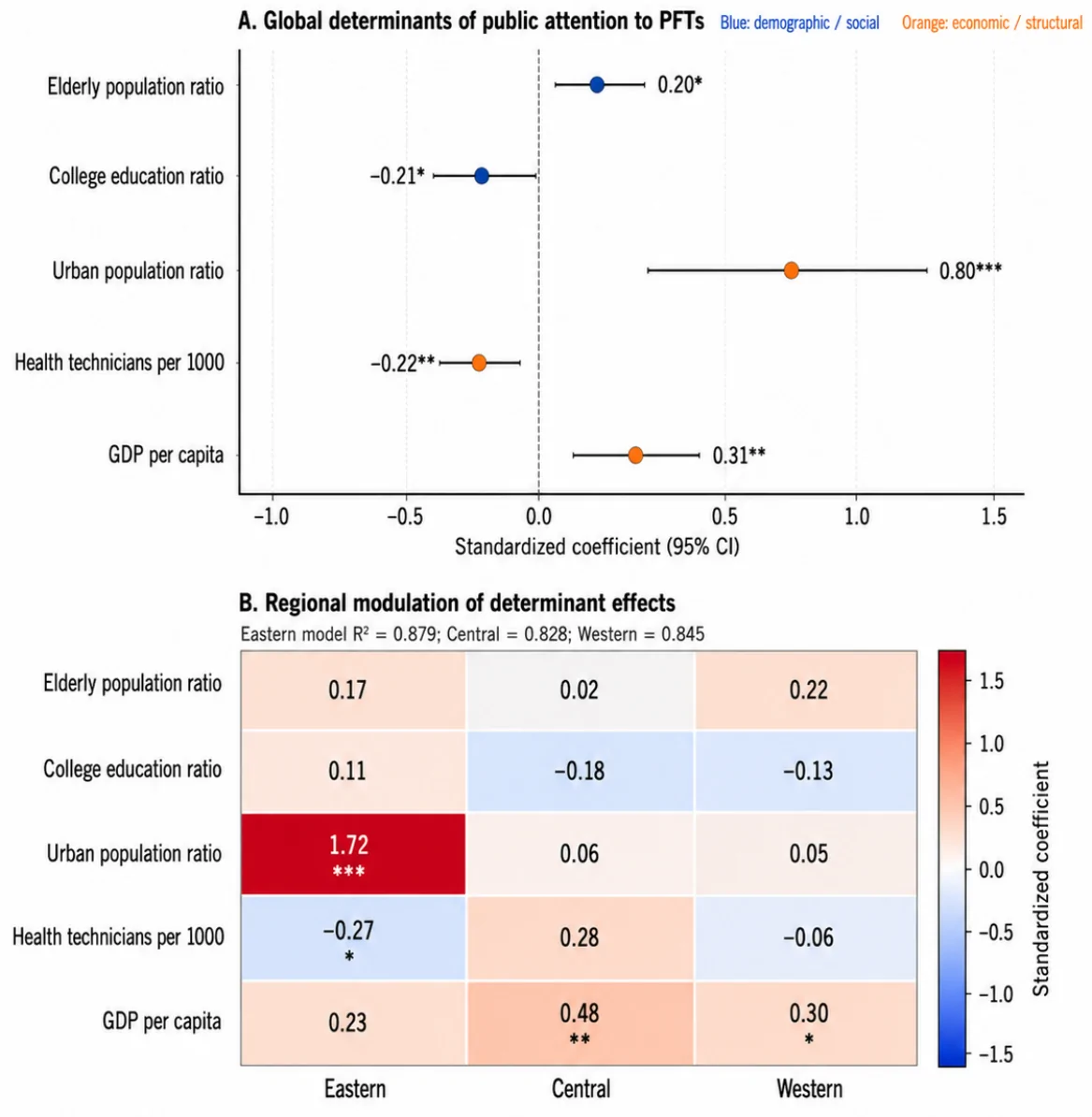
Socioeconomic factors associated with PFT-related Baidu Index values and subgroup heterogeneity in China. (**A**) Standardized coefficients from the fixed-effects panel regression model estimating the association between socioeconomic determinants and pulmonary function testing-related search activity. Points indicate standardized coefficients, and horizontal lines indicate 95% confidence intervals. Blue markers represent demographic or social variables, and orange markers represent economic or structural variables. (**B**) Regional heterogeneity in determinant effects across Eastern, Central, and Western China. Cell values indicate standardized coefficients from region-specific models, and the color scale represents the direction and magnitude of the association. Asterisks denote statistical significance.

Figure 6A presents the standardized associations between socioeconomic variables and PFT-related Baidu Index values in the fixed-effects panel regression, and Table 3 provides the complete numerical estimates. In the full model, per capita GDP was positively associated with PFT-related Baidu Index values (unstandardized coefficient = 0.0476, 95% CI: 0.0155 to 0.0797, standardized coefficient = 0.31, p=0.004). Urban population ratio also showed a positive association (unstandardized coefficient = 324.81, 95% CI: 137.53 to 512.09, standardized coefficient = 0.80, p<0.001). The elderly population ratio was positively associated with PFT-related search intensity (unstandardized coefficient = 30,029.50, 95% CI: 6697.59 to 53,361.42, standardized coefficient = 0.20, p=0.012). In contrast, the college education ratio was negatively associated with PFT-related Baidu Index values (unstandardized coefficient = −13,423.57, 95% CI: −16,552.54 to −194.59, standardized coefficient = −0.21, p=0.045), and health technicians per 1000 population also showed a negative association (unstandardized coefficient = −573.11, 95% CI: −997.61 to −148.62, standardized coefficient = −1.22, p=0.008). These findings describe ecological associations at the provincial level and should not be interpreted as causal effects or as direct evidence of individual healthcare-seeking behavior.

In the regional subgroup models shown in Figure 6B, urbanization rate showed marked geographic heterogeneity. The standardized coefficient for urbanization rate was much larger in Eastern China (standardized coefficient = 1.72) than in Central China (standardized coefficient = 0.06) or Western China (standardized coefficient = 0.05). This pattern suggests that the association between urbanization and PFT-related Baidu Index values was mainly observed in eastern provinces, although this subgroup result should be interpreted as exploratory because formal causal interaction effects were not established.

The subgroup analyses showed heterogeneity in the association between population aging and PFT-related Baidu Index values. As shown in Figure 6B and Table 4, the standardized coefficient for the elderly population ratio was positive but not statistically significant in Eastern China (standardized coefficient = 0.17, 95% CI: −1.06 to 0.40, p=0.142), Central China (standardized coefficient = 0.02, 95% CI: −1.54 to 0.57, p=0.954), and Western China (standardized coefficient = 0.22, 95% CI: −1.11 to 0.55, p=0.181). By income subgroup, the association was weak and non-significant in high-income provinces (standardized coefficient = 0.06, 95% CI: −1.15 to 0.28, p=0.561), but positive and statistically significant in low-income provinces (standardized coefficient = 0.39, 95% CI: 0.14 to 0.64, p=0.003). A similar pattern was observed by urbanization level: the association was not statistically significant in high-urbanization provinces (standardized coefficient = 0.01, 95% CI: −1.20 to 0.22, p=0.951), but was positive and statistically significant in low-urbanization provinces (standardized coefficient = 0.42, 95% CI: 0.14 to 0.71, p=0.004). These subgroup differences should be interpreted as heterogeneous ecological associations rather than evidence of specific behavioral or healthcare mechanisms.

**Figure 7:**
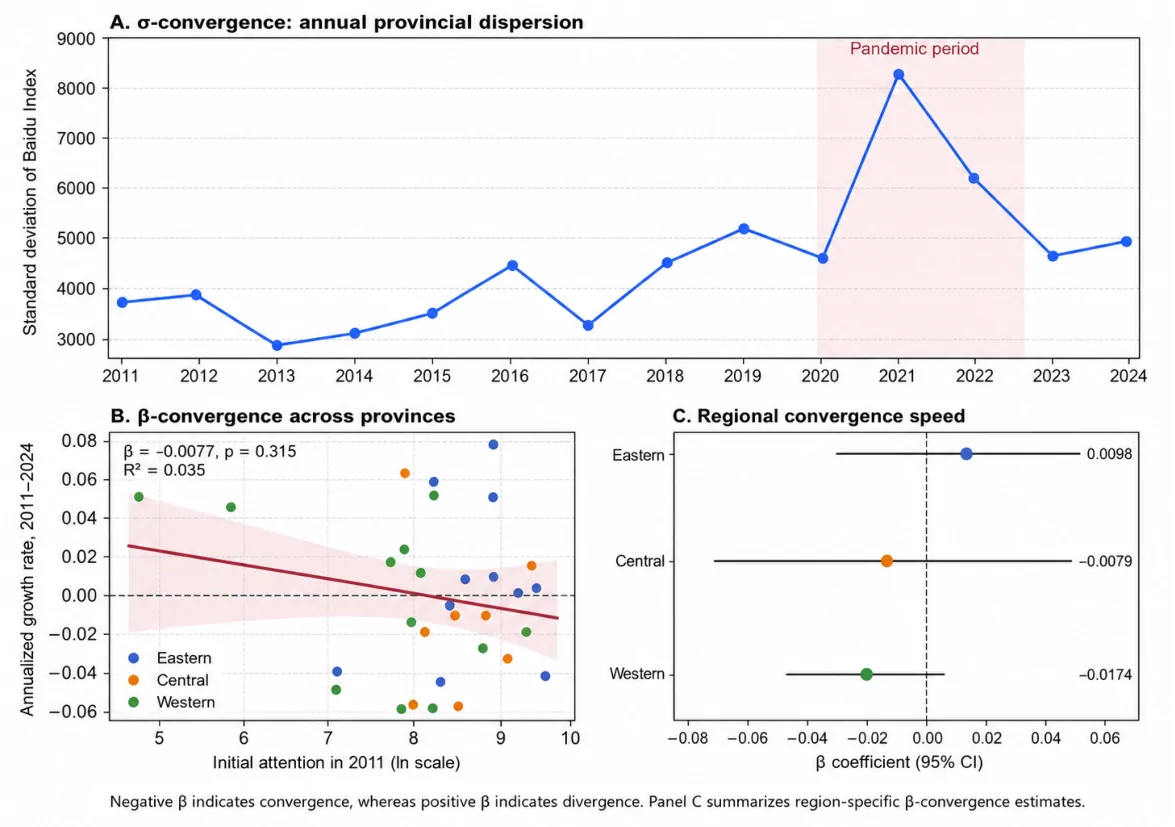
Spatial convergence dynamics of public attention to pulmonary function testing. (**A**) σ-convergence analysis showing annual provincial dispersion in pulmonary function testing-related Baidu Index values. (**B**) β-convergence analysis across provinces, showing the association between initial attention in 2011 and annualized growth from 2011 to 2024. The red line represents the fitted ordinary least-squares regression line for the β-convergence model, and the shaded band indicates the 95% confidence interval of the fitted line. A negative β coefficient indicates convergence, whereas a positive β coefficient indicates divergence. (**C**) Region-specific β-convergence estimates for Eastern, Central, and Western China. Points represent estimated β coefficients, and horizontal lines indicate 95% confidence intervals. The shaded area in Panel A denotes the COVID-19 pandemic period.

Figure 7 and Table 2, Panel B summarize the spatial convergence results. In the full-sample β-convergence model, the association between initial PFT-related attention in 2011 and annualized growth from 2011 to 2024 was negative but not statistically significant (β=−0.0077, 95% CI: −1.0230 to 0.0077, p=0.315, $R^{2}=0.035$). Region-specific analyses showed a positive but non-significant estimate in Eastern China (β=0.0098, 95% CI: −1.0367 to 0.0563, p=0.645, $R^{2}=0.025$), a negative but non-significant estimate in Central China (β=−0.0079, 95% CI: −1.0819 to 0.0661, p=0.802, $R^{2}=0.011$), and a negative but non-significant estimate in Western China (β=−0.0174, 95% CI: −1.0384 to 0.0036, p=0.095, $R^{2}=0.253$). These results provide limited statistical evidence of β-convergence in PFT-related Baidu Index values across provinces.

## 4. Discussion

This study examined Baidu-based online search activity related to the selected PFT-related keyword in China from 2011 to 2024. PFT-related Baidu Index values were consistently lower than those for blood pressure and glucose monitoring. Because Baidu Index reflects platform-specific online search behavior rather than clinical service utilization, this finding should be interpreted as lower relative search intensity within the Baidu ecosystem, not as direct evidence of PFT underuse, unmet clinical need, inadequate public recognition, or individual-level health behavior. Nevertheless, spirometry is clinically important for the diagnosis and management of COPD and asthma [5,6], and previous studies have shown that spirometry remains underused in clinical practice [7,8,9,10,11,17]. Together with prior infodemiology studies showing that search engine data can reflect population-level health information seeking [12,13,14,18,19,20], our findings may generate the hypothesis that PFT-related information is less frequently searched than more familiar chronic disease monitoring topics within the Baidu ecosystem. The comparison with blood pressure and glucose monitoring should be interpreted cautiously. These comparator terms were selected because they represent familiar chronic disease monitoring tools and provide a descriptive benchmark for relative Baidu search intensity. However, they are not conceptually equivalent to pulmonary function testing. Blood pressure and glucose monitoring are commonly performed at home, whereas spirometry and other pulmonary function tests are usually conducted in clinical or professionally supervised settings. Consequently, lower PFT-related search intensity may reflect differences in keyword meaning, device ownership, testing setting, cost, accessibility, clinical indications, and user composition, rather than lower public recognition alone.

The temporal pattern observed during 2020–2022 may reflect the broader public health context of COVID-19, during which respiratory health topics became more salient in online information environments. Previous infodemiology studies have shown that internet search data can capture population responses to emerging health threats and complement traditional public health surveillance [18,19,20,21]. In addition, health behavior frameworks suggest that perceived severity, vulnerability, response efficacy, and self-efficacy may influence protective behaviors and health information seeking [22,23,24]. However, the re-specified ITSA using unsmoothed monthly observations and seasonality adjustment did not provide strong statistical evidence for an immediate level change or sustained slope change. Therefore, the pandemic-period pattern should be interpreted cautiously as an observed temporal association in Baidu-based search activity, rather than definitive evidence that COVID-19 increased respiratory health awareness, changed PFT-related behavior, or increased clinical testing uptake. Media exposure, platform behavior, changes in health information needs, and other contemporaneous factors may also have contributed to the observed search patterns.

Although PFT-related Baidu search activity was descriptively higher during 2020–2022, this pattern was not sufficient to establish a durable change in public recognition, respiratory-health literacy, or clinical testing behavior. The search activity observed after the policy transition also remained lower than that for blood pressure and glucose monitoring, but this comparison should be interpreted only as lower relative Baidu search intensity for the selected keyword. It should not be interpreted as evidence of a fixed hierarchy of public health demand or as proof of a persistent recognition gap. These findings support the potential value of routine respiratory health communication, but they do not demonstrate that pandemic-related online search patterns translated into actual PFT uptake. Future health communication strategies may consider integrating PFT-related information into chronic disease prevention and respiratory health promotion, particularly for older adults and high-risk populations [17].

Another major finding is the persistent provincial variation in PFT-related Baidu Index values. The Gini coefficient, Theil index, and coefficient of variation showed that provincial differences fluctuated over time but did not disappear. The fixed-effects and subgroup analyses suggested that population aging was positively associated with PFT-related Baidu Index values, with stronger exploratory associations in low-income and low-urbanization subgroups. This pattern is compatible with the higher burden of chronic respiratory disease among older populations [1,2,3,4]. However, these are ecological associations based on provincial-level Baidu search data. They should not be interpreted as evidence that older adults themselves conducted the searches, nor as proof that population aging causally increased PFT use, spirometry knowledge, public recognition, or respiratory-health literacy. Searches may also have been conducted by caregivers, healthcare workers, students, institutional purchasers, or commercial users. In addition to population aging, urbanization showed notable regional heterogeneity. The association between urbanization rate and PFT-related Baidu Index values was much stronger in Eastern China than in Central or Western China. This pattern may reflect the fact that urbanization in eastern coastal provinces is more closely linked to higher digital connectivity, greater concentration of healthcare resources, more frequent institutional health screening, and more active online health information seeking. By contrast, in central and western regions, the relationship between urbanization and Baidu-based PFT-related search activity may be attenuated by differences in digital access, healthcare-service availability, population distribution, or local information-seeking pathways. However, these explanations were not directly tested and should be interpreted as hypotheses for future research rather than causal conclusions.

Prior studies have shown that e-health information-seeking behavior among older adults may be influenced by perceived usefulness, self-efficacy, access barriers, and digital literacy [25]. However, these individual-level mechanisms were not directly measured in the present ecological study. Similarly, the negative associations involving education level and health workforce density should be interpreted only as ecological associations. They should not be taken as evidence of substitution effects, lower awareness, reduced respiratory-health literacy, or specific healthcare-seeking mechanisms. Online health information seeking is shaped by education, income, digital literacy, trust, accessibility, and information quality [26,27]; therefore, Baidu Index reflects only one platform-specific component of health information behavior and may under-represent groups who obtain information through clinical consultation, institutional health resources, professional platforms, social media, or other digital platforms.

The convergence analysis further suggests that provincial differences in PFT-related Baidu Index values persisted over time. The weak and statistically non-significant β-convergence results indicate that provinces with initially lower PFT-related Baidu Index values did not systematically catch up with provinces with higher initial values. Although σ-dispersion fluctuated during the pandemic period, there was no strong evidence of stable long-term convergence. These findings should be interpreted as patterns of platform-specific search dispersion, not as direct evidence of convergence or divergence in clinical service use, respiratory health needs, or public recognition. From a health communication perspective, digital platforms may be useful for disseminating respiratory health information, but future studies combining search data with clinical utilization records, survey-based health literacy measures, and multi-platform digital traces are needed to determine whether online search patterns are associated with actual PFT uptake or respiratory health outcomes.

Several limitations should be acknowledged. First, Baidu Index reflects platform-specific online search behavior rather than actual clinical utilization of PFTs; therefore, it should be interpreted as a proxy for Baidu-based search interest in the selected keyword, not as a direct measure of testing uptake, clinical demand, spirometry knowledge, respiratory-health literacy, disease burden, or unmet healthcare need. Second, keyword-based analysis may not capture all semantic variations in respiratory health searches. The PFT/BP and PFT/glucose ratios also have a construct-validity limitation because PFT-related instrument searches may reflect product information seeking, purchasing interest, institutional procurement, or commercial attention, while blood pressure and glucose monitoring are commonly home-based self-monitoring behaviors and pulmonary function testing is usually a clinical or professionally supervised procedure. Thus, the lower ratios may partly reflect differences in keyword meaning, testing modality, accessibility, care pathways, and user composition rather than lower public recognition alone. Third, Baidu-based search patterns may have been influenced by changes in China’s digital information ecosystem, including the increasing use of WeChat, Douyin, and Xiaohongshu for health information seeking. Such platform migration may vary by age group and region, potentially affecting long-term trend estimation and regional convergence analysis. Fourth, internet search data may also be affected by media coverage, algorithm changes, regional internet penetration, digital access, and Baidu platform use, which are common limitations of search-based infodemiology studies [18]. Finally, the ecological design limits individual-level causal inference. Future studies should combine search engine data with clinical service utilization, modality-equivalent appointment-related search terms, survey-based health literacy measures, and multi-platform digital trace data to evaluate whether Baidu-based online search interest is associated with actual testing behavior, PFT uptake, and respiratory health outcomes.

Overall, this study describes long-term Baidu-based online search patterns for the selected PFT-related keyword in China. PFT-related Baidu search activity remained lower than search activity for blood pressure and glucose monitoring, and provincial differences persisted over time. These findings may suggest that PFT-related information is less visible within the Baidu-based online information environment, but they should not be interpreted as direct evidence of clinical underuse, unmet respiratory healthcare need, inadequate public recognition, respiratory-health literacy, or individual-level behavior. Sustained respiratory health communication and targeted digital health strategies may be useful, but future studies using clinical utilization data, survey measures, and multi-platform digital traces are needed to determine whether online search interest is associated with actual PFT uptake and respiratory health outcomes.

## 5. Conclusions

This 14-year Baidu Index-based study showed that PFT-related Baidu search activity in China remained lower than search activity for blood pressure and glucose monitoring from 2011 to 2024. Descriptive increases were observed during the pandemic period, but the re-specified seasonality-adjusted ITSA did not provide strong statistical evidence for an immediate level change or sustained slope change. Provincial differences in PFT-related Baidu Index values persisted over time, and β-convergence analysis provided limited statistical evidence that initially low-attention provinces systematically caught up with high-attention provinces. Population aging was positively associated with PFT-related Baidu Index values, particularly in some less developed regional subgroups, but this should be interpreted as an ecological association rather than a causal driver.

These findings suggest that PFT-related information may be relatively less visible than blood pressure and glucose monitoring information within the Baidu-based online information environment. However, lower Baidu search activity should not be interpreted as direct evidence of clinical underuse, unmet respiratory healthcare need, or inadequate public recognition. Future studies should combine search engine data with clinical utilization records, appointment-related search terms, survey-based health literacy measures, and multi-platform digital trace data to determine whether online attention is associated with actual PFT uptake and respiratory health outcomes.

## Figures and Tables

**Table 1:** Primary interrupted time-series estimates for PFT-related Baidu Index values.

Panel	Parameter	Definition	Estimate	SE	95% CI	*p*-Value
**Panel A. Segmented regression coefficients**						
A	$\beta_{1}$	Pre-pandemic slope	0.12	0.10	−0.08 to 0.33	0.237
A	$\beta_{2}$	Immediate level change at pandemic onset	8.19	19.62	−30.58 to 46.96	0.677
A	$\beta_{3}$	Pandemic-period slope change	1.97	1.12	−0.25 to 4.20	0.081
A	$\beta_{4}$	Immediate level change at post-transition onset	−17.19	17.69	−52.13 to 17.76	0.333
A	$\beta_{5}$	Post-transition slope change	1.35	1.13	−0.88 to 3.58	0.233
**Panel B. Phase-specific monthly slopes**						
B	–	Pre-pandemic slope	0.12	0.10	−0.08 to 0.33	0.237
B	–	Pandemic-period slope	2.10	1.12	−0.11 to 4.30	0.062
B	–	Post-transition slope	1.48	1.12	−0.74 to 3.69	0.191

*Note:* The model used 168 unsmoothed monthly observations from January 2011 to December 2024. Calendar-month fixed effects were included to adjust for seasonality. Newey–West robust standard errors with a lag length of 12 were used. The post-transition period began in January 2023.

**Table 2:** Spatial inequality and convergence estimates for PFT-related Baidu Index values.

Panel A. Spatial Inequality Indices					
Period	Gini coefficient	Theil index	Within-region Theil	Between-region Theil	CV
Pre-pandemic, 2011–2019	0.443 ± 0.043	0.331 ± 0.065	0.231 ± 0.046	0.100 ± 0.037	0.832 ± 0.094
Pandemic, 2020–2022	0.365 ± 0.047	0.222 ± 0.055	0.156 ± 0.032	0.065 ± 0.023	0.656 ± 0.085
Post-pandemic, 2023–2024	0.450 ± 0.003	0.333 ± 0.006	0.225 ± 0.018	0.108 ± 0.013	0.846 ± 0.009
**Panel B. β-Convergence Analysis**					
Model	β estimate	95% CI	*p*-value	$R^{2}$	
Full sample	−0.0077	−0.0230 to 0.0077	0.315	0.035	
Eastern China	0.0098	−0.0367 to 0.0563	0.645	0.025	
Central China	−0.0079	−0.0819 to 0.0661	0.802	0.011	
Western China	−0.0174	−0.0384 to 0.0036	0.095	0.253	

*Note:* Values in Panel A are presented as mean ± SD across years within each period. CV denotes coefficient of variation. β-convergence was estimated by regressing annualized log growth from 2011 to 2024 on the log-transformed initial Baidu Index value in 2011. A negative β estimate indicates convergence, whereas a positive β estimate indicates divergence.

**Table 3:** Two-way fixed-effects regression of socioeconomic factors associated with PFT-related Baidu Index values.

Variable	Unstandardized Coefficient	Robust SE	95% CI	Standardized Coefficient	Robust SE	*p*-Value
GDP per capita	0.0476	0.0163	0.0155 to 0.0797	0.31	0.11	0.004
Urban population ratio	324.81	95.24	137.53 to 512.09	0.80	0.24	<0.001
College education ratio	−13,423.57	6676.96	−26,552.54 to −294.59	−1.21	0.10	0.045
Elderly population ratio	30,029.50	11,865.84	6697.59 to 53,361.42	0.20	0.08	0.012
Health technicians per 1000	−173.11	215.88	−197.61 to −148.62	−1.22	0.08	0.008

*Note:* The dependent variable was the provincial annual PFT-related Baidu Index value. Province and year fixed effects were included. Robust standard errors were calculated using HC1 heteroskedasticity-consistent estimation. Standardized coefficients were obtained by standardizing both dependent and independent variables. Complete-case analysis was used because health workforce density had missing values in some province-year observations. Number of observations = 424; number of provinces = 31; study years = 2011–2024; $R^{2}=0.847$; adjusted $R^{2}=0.827$.

**Table 4:** Subgroup regression summary for the association between population aging and PFT-related Baidu Index values.

Subgroup	Standardized Coefficient	Robust SE	95% CI	*p*-Value	Observations	Adjusted $R^{2}$
Eastern China	0.17	0.11	−1.06 to 0.40	0.142	150	0.851
Central China	0.02	0.28	−1.54 to 0.57	0.954	110	0.777
Western China	0.22	0.17	−1.11 to 0.55	0.181	164	0.811
High-income provinces	0.06	0.11	−1.15 to 0.28	0.561	219	0.844
Low-income provinces	0.39	0.13	0.14 to 0.64	0.003	205	0.776
High-urbanization provinces	0.01	0.11	−1.20 to 0.22	0.951	219	0.852
Low-urbanization provinces	0.42	0.14	0.14 to 0.71	0.004	205	0.771

*Note:* Each subgroup model included province and year fixed effects and the same covariates as the main fixed-effects model. Coefficients are standardized within each subgroup. Robust standard errors were calculated using HC1 estimation. Subgroup findings should be interpreted as heterogeneous ecological associations rather than causal effects.

## Data Availability

The raw data supporting the conclusions of this article will be made available by the authors on request.

## Supplementary Materials

The following supporting information can be downloaded at: https://www.mdpi.com/article/10.3390/healthcare14162640/s1, Table S1. Interrupted time-series model diagnostics and sensitivity analyses; Table S2. Annual spatial inequality and sigma-dispersion indices, 2011–2024; Table S3. Full subgroup fixed-effects regression coefficients; Table S4. Annual keyword-level Baidu Index values and relative search-intensity ratios.

## Abbreviations

The following abbreviations are used in this manuscript:

BI	Baidu Index
BP	Blood pressure
COPD	Chronic obstructive pulmonary disease
FVC	Forced vital capacity
FEV_1_	Forced expiratory volume in one second
GDP	Gross domestic product
ITSA	Interrupted time series analysis
OLS	Ordinary least squares
PFT	Pulmonary function test
VIF	Variance inflation factor
